# Development and Evaluation of Knowledge-Based Treatment Plans for Chest Wall and Regional Lymph Node Irradiation

**DOI:** 10.7759/cureus.100934

**Published:** 2026-01-06

**Authors:** Panagiota Galanakou, Nesrin Dogan, Stuart E Samuels, Maria De La Luz De Ornelas, Robert Kaderka

**Affiliations:** 1 Department of Radiation Oncology, University of Miami, Miami, USA

**Keywords:** automated treatment planning, automated vmat, chest wall with lymph nodes radiotherapy, knowledge-based treatment planning, radiotherapy treatment planning

## Abstract

Background

Knowledge-based planning (KBP) improves radiotherapy efficiency and consistency by using machine learning models trained on prior high-quality plans. Most breast KBP studies focus on whole- or partial-breast treatments without nodal coverage, and limited attention has been given to laterality-specific chest wall volumetric modulated arc therapy (VMAT) plans. This study develops left-sided, right-sided, and combined KBP models for chest wall and regional nodal irradiation and compares their performance with clinical plans.

Materials and methods

KBP models were created using 47 left-sided and 44 right-sided chest wall patients involving regional lymph nodes. A combined model incorporating all cases was also developed. Optimization objectives were iteratively refined using model-predicted, manual, and normal tissue objectives (NTOs). Model performance was evaluated using the coefficient of determination (R²), chi-square (χ²), and mean squared error (MSE). For validation, 10 left-sided, 10 right-sided, and 20 combined KBP plans were compared with their corresponding clinical plans using paired t-tests (p < 0.05). Dosimetric endpoints included target coverage, conformity index, homogeneity index, organ-at-risk (OAR) dose-volume metrics, and delivery efficiency factor (intensity modulated radiation therapy factor). KBP plans were generated without planner intervention, with additional optimization using a monitor unit objective when required. All plans underwent blinded physician review for clinical acceptability and preference.

Results

Left-sided KBP plans significantly reduced mean doses to the esophagus (-499 ± 147 cGy, p < 0.05) and thyroid (-296 ± 96 cGy, p < 0.05), with small increases in spinal cord maximum dose (20 ± 82 cGy, p > 0.05) and V15% of the contralateral lung (10.1 ± 1.9, p < 0.05). Right-sided KBP plans reduced mean doses to the thyroid (-220 ± 86 cGy, p < 0.05) and heart (-46.1 ± 9.3 cGy, p < 0.05), with minimal impact on coverage of the planning target volume of the internal mammary nodes (PTV_IMN). The combined model demonstrated similar dosimetric patterns. KBP plans improved conformity in six of 10 cases and homogeneity in 50% of cases, achieving higher dose values corresponding to 98% of the prescription dose (D98%) in 60-65% of cases for laterality-specific and combined KBP models, respectively, while maintaining the maximum dose (Dmax) and the dose received by 105% of the prescription dose (D105%) within constraints. Delivery efficiency remained comparable. A blinded physician review favored KBP in the majority of cases, with equivalence noted in others.

Conclusions

Laterality-specific and combined KBP models for VMAT chest wall and nodal irradiation generate plans that are dosimetrically comparable or superior to manual plans, providing consistent target coverage and improved or maintained OAR sparing. KBP offers a reproducible and efficient strategy for complex breast and chest wall cases, supports workflow standardization, and requires only minimal refinement in selected situations.

## Introduction

Knowledge-based planning (KBP) has emerged as a powerful approach in radiotherapy to improve both planning efficiency and treatment consistency. Using machine learning techniques, KBP models are trained on prior high-quality treatment plans to predict achievable dose-volume histogram (DVH) objectives and optimization parameters for new patients. This data-driven methodology allows institutional planning experience to be translated into automated model guidance, thereby reducing inter-planner variability, streamlining plan generation, and promoting consistent plan quality across diverse patient anatomies [1,2].

KBP has been explored in breast radiotherapy; however, most published work has focused on whole-breast or partial-breast irradiation without regional nodal coverage. For example, Apaza Blanco et al. [3] reported separate volumetric modulated arc therapy (VMAT) KBP models for left- and right-sided whole-breast treatments and demonstrated more consistent plan quality compared with manual planning, although their approach did not include nodal regions. Other investigations have applied KBP for intensity modulated radiation therapy (IMRT) for partial-breast irradiation, showing reduced planning variability and greater efficiency in localized treatment settings [4]. More recently, both IMRT [5] and VMAT KBP models [6] have been developed for post-mastectomy chest wall irradiation involving the internal mammary chain and supraclavicular fossa. These studies demonstrate the feasibility of KBP in more complex locoregional scenarios and suggest potential dosimetric benefits, particularly in reducing low-dose exposure to surrounding organs at risk (OARs).

Despite these advances, relatively limited attention has been given to laterality-specific VMAT chest wall models that comprehensively include regional nodal irradiation (RNI). The dosimetric trade-offs differ substantially between left- and right-sided treatments: for left-sided cases, minimizing cardiac dose is paramount, whereas for right-sided cases, sparing of the contralateral lung and breast is more critical. Whether separate laterality-specific models provide advantages over a single unified model has not been systematically evaluated, despite important implications for clinical implementation and workflow standardization.

This study addresses these gaps by developing and validating two laterality-specific KBP models, one for left-sided and one for right-sided chest wall and nodal irradiation, along with a unified combined model. Their performance is compared with clinically generated treatment plans in terms of OAR sparing and overall plan quality. In addition, a blinded physician review is conducted to evaluate clinical acceptability and preference between KBP-generated and manually created plans.

Taken together, this work aims to clarify the relative strengths of separate versus combined KBP models and to provide practical guidance for implementing KBP in complex chest wall and nodal radiotherapy. To our knowledge, no prior KBP studies have directly evaluated this question in chest wall irradiation with comprehensive regional nodal coverage.

## Materials and methods

Patient selection and clinical treatment planning

This retrospective study was conducted in the Department of Radiation Oncology at the University of Miami (Miami, USA) and included patients treated between 2016 and 2024. Two separate KBP models were developed using clinical datasets from 91 patients who previously received chest wall and regional lymph node irradiation for breast cancer. The cohort included 47 left-sided and 44 right-sided cases. All patients were treated in the supine position using either free-breathing or deep-inspiration breath-hold techniques [7,8].

Patients without regional nodal involvement were excluded. The study cohort consisted of patients who received postmastectomy chest wall irradiation with comprehensive nodal coverage. RNI included the supraclavicular, axillary, and internal mammary lymph node regions. To maintain consistency with VMAT planning, patients treated with IMRT or conformal 3D techniques were excluded. Boost treatments were not considered in this analysis.

The treatment plans and contours used for model development were derived from routine clinical practice and reflect contributions from multiple dosimetrists and radiation oncologists over the study period. This introduced natural variability in target and OAR delineation, as well as in planning strategies, providing a heterogeneous dataset for model training. Such diversity was considered beneficial, as it enhances the generalizability of the KBP models to future patients with varying anatomical geometries and clinical planning preferences.

Target volumes were defined by physicians specializing in breast cancer, following the Radiation Therapy Oncology Group (RTOG) contouring guidelines. Clinical target volumes (CTVs) included the chest wall, any undissected axillary nodes, supraclavicular and infraclavicular nodes, and internal mammary nodes. Planning target volumes (PTVs) were generated by adding a 5-mm margin to the respective CTVs and were cropped 3 mm inside the body contour to exclude skin. The OARs considered in this study included the heart, ipsilateral lung, contralateral lung, contralateral breast, esophagus, spinal cord, and thyroid.

Clinical treatment planning was performed using the Eclipse treatment planning system (v15.0 and v16.1; Varian Medical Systems, Inc., Palo Alto, CA, USA) with the VMAT technique, the Photon Optimizer optimization algorithm, and the AcurosXB dose calculation algorithm (Varian Medical Systems, Inc.), using a 0.25-cm dose grid in all cases. Three to four partial arcs with 6-MV photon beams were selected based on the complexity of the target volumes. In this study, all VMAT plans were generated using a standard tangential approach. Other VMAT configurations, including butterfly or bowtie techniques, have been explored in previous KBP models and studies [2,9-11].

The prescription dose to the PTV was either 42.56 Gy in 16 fractions or 40.05 Gy in 15 fractions. Plans were normalized to ensure that ≥95% of the total PTV received ≥95% of the prescribed dose. The total PTV (PTVTotal) included the chest wall (PTVChestwall), supraclavicular nodes (PTVSupraclavicular), axillary nodes (PTVAxilla), and internal mammary nodes (PTVIMN). Coverage criteria were as follows: PTVChestwall and PTVSupraclavicular were required to receive ≥95% of the prescribed dose to ≥95% of their volumes, whereas PTVIMN was required to receive ≥90% of the prescribed dose to ≥90% of its volume. Treatments were delivered on Trilogy, Edge, or TrueBeam linear accelerators.

Model creation 

A total of 47 left-sided and 44 right-sided previously treated chest wall patients with regional lymph node involvement were used to create three KBP models: a left-sided model (KBP_LT_), a right-sided model (KBP_RT_), and a combined model (KBP_c_). All models were developed using RapidPlan (Eclipse v16.1, Varian Medical Systems, Inc.). Geometric and dosimetric outliers within the training datasets were identified as suggested in [12] and excluded to enhance model robustness. This process is often referred to as quality filtering, in which a secondary training phase was performed on the refined dataset to further improve model accuracy [12-14].

The optimization objectives were iteratively adjusted to align with institutional clinical planning standards, integrating model-generated objectives, manual planning constraints, and automated normal tissue sparing goals. An iterative tuning process was employed in which KBP-generated plans were compared with their corresponding clinical plans. In this tuning process, six plans were used for each model. If the model underperformed in any dosimetric aspect, the optimization objectives were adjusted, and the process was repeated until all plans met acceptable clinical standards. The overarching goal was to develop models capable of producing plans that matched or exceeded the quality of the original clinical plans.

The final optimization objectives for the left-sided, right-sided, and combined models are summarized in Table 1 and Table 2. The “line” objective refers to the optimization constraint that prioritizes OAR sparing in regions overlapping with target volumes. A generalized equivalent uniform dose objective, with a parameter a = 40, was used to limit high-dose regions within OARs by constraining the maximum EUD that a structure could receive. Additionally, a manual NTO was applied to achieve a sharp dose fall-off around the targets and minimize dose to adjacent healthy tissues. The NTO parameters were set as follows: distance from target border = 0.5 cm, start dose = 100%, end dose = 50%, and fall-off = 0.20.

**Table 1:** Optimization objectives for the KBPLT and KBPc chest wall with LNs models The total planning target volume (PTVTotal) includes the axillary nodes planning target volume (PTV_Axilla), the chest wall planning target volume (PTV_Chestwall), the internal mammary nodes planning target volume (PTV_IMN), and the supraclavicular nodes planning target volume (PTV_Supraclav). A_LAD, left anterior descending coronary artery; gEUD, generalized equivalent uniform dose; KBP_c_, combined laterality knowledge-based planning; KBP_LT_, left-sided knowledge-based planning; LN, lymph node

Structure	Type	Vol (%)	Dose	Priority
PTVTotal	Upper	0	103%	160
	Upper	0.1	104%	100
	Lower	96	100%	300
PTV_Axilla	Upper	0	103%	150
	Upper	0.1	102%	100
	Lower	96	100%	300
PTV_Chestwall	Upper	0	103%	160
	Upper	0.1	101.40%	100
	Lower	100	97%	300
PTV_IMN	Upper	0	103%	150
	Upper	0.1	102%	100
	Lower	95	95%	300
PTV_Supraclav	Upper	0	103%	150
	Upper	0.1	102%	100
	Lower	100	96%	300
Heart	Mean		115 cGy	250
	gEUD		40%	100 (a = 40)
	Line	Generated	Generated	100
A_LAD	Upper	100	40%	100
	Mean		Generated	49
	gEUD		40%	100 (a = 40)
	Line	Generated	Generated	50
Lung ipsilateral	Upper	25	11%	160
	Upper	20	22%	160
	Upper	15	45%	160
	Upper	Generated	500 cGy	49
	Upper	Generated	1000 cGy	49
	Upper	Generated	2000 cGy	49
	Mean	Generated		99
	Line	Generated	Generated	50
Lung contralateral	Mean		Generated	239
	Line	Generated	Generated	70
Breast contralateral	Upper	10	7%	250
	Upper	1	7%	200
	Mean		Generated	50
Thyroid	Mean		Generated	90
	Mean		37%	150
	Line	Generated	Generated	60
Esophagus	Mean		1800 cGy	100
	gEUD		2500 cGy	200 (a = 40)
	Line	Generated	Generated	80
Spinal cord	Upper	0	16%	60
	Upper	0	25%	100
	Upper	0	Generated	60
Humerus LT/RT	Upper	Generated	57%	49
Larynx	Mean		Generated	49
	Line	Generated	Generated	50
Trachea	Mean		Generated	50
Brachial plexus LT/RT	Line	Generated	Generated	50

**Table 2:** Optimization objectives for the KBPRT chest wall with LNs model The total planning target volume (PTVTotal) includes the axillary nodes planning target volume (PTV_Axilla), the chest wall planning target volume (PTV_Chestwall), the internal mammary nodes planning target volume (PTV_IMN), and the supraclavicular nodes planning target volume (PTV_Supraclav). A_LAD, left anterior descending coronary artery; gEUD, generalized equivalent uniform dose; KBP_RT_, right-sided knowledge-based planning; LN, lymph node

Structure	Type	Vol (%)	Dose	Priority
PTVTotal	Upper	0	103%	160
	Upper	0.1	104%	100
	Lower	96	100%	300
PTV_Axilla	Upper	0	103%	150
	Upper	0.1	102%	100
	Lower	98	100%	300
PTV_Chestwall	Upper	0	103%	160
	Upper	0.1	101.40%	100
	Lower	100	97%	300
PTV_IMN	Upper	0	103%	150
	Upper	0.1	102%	100
	Lower	95	95%	300
PTV_Supraclav	Upper	0	103%	150
	Upper	0.1	102%	100
	Lower	100	97%	300
Heart	Mean		150 cGy	255
	gEUD		40%	100 (a = 40)
	Line	Generated	Generated	100
A_LAD	Upper	100	40%	100
	Mean		Generated	49
	gEUD		40%	100 (a = 40)
	Line	Generated	Generated	50
Lung ipsilateral	Upper	25	11%	160
	Upper	20	22%	160
	Upper	15	45%	160
	Upper	Generated	500 cGy	49
	Upper	Generated	1000 cGy	49
	Upper	Generated	2000 cGy	49
	Mean	Generated		99
	Line	Generated	Generated	50
Lung contralateral	Upper	5	400	180
	Mean	NA	Generated	239
Breast contralateral	Upper	10	7%	250
	Upper	1	7%	200
	Upper	5	450 cGy	169
	Line	Generated	Generated	50
Thyroid	Mean		Generated	49
	Mean		37%	100
	Line	Generated	Generated	60
Esophagus	Mean		Generated	49
	Line	Generated	Generated	50
Spinal cord	Upper	0	16%	60
	Upper	0	25%	100
	Upper	0	Generated	60
Humerus LT/RT	Upper	Generated	57%	49
Larynx	Mean		Generated	49
	Line	Generated	Generated	50
Trachea	Mean		Generated	50
Brachial plexus LT/RT	Line	Generated	Generated	50

To control the maximum dose (Dmax), an upper objective of 103% of the prescription dose was applied with a priority of 1000. In addition, a monitor unit (MU) objective was introduced, limiting the maximum number of MUs to three times the dose per fraction. Both the Body Max and MU objectives were incorporated during the secondary optimization run, beginning at optimization level 3, to further refine dose distribution and enhance plan efficiency.

KBP performance and model validation

The performance of each KBP model was assessed using standard statistical metrics [2], including the coefficient of determination (R²), chi-square (χ²), and mean squared error (MSE) of the overall estimation.

The R² value represents the proportion of variance in the dependent variable (DVH principal component score) that can be explained by the independent variable (geometric features). Values close to 1.0 indicate a strong predictive relationship and good model performance. However, R² values greater than 1 may suggest overfitting, where the model describes the training data well but loses generalizability to new cases.

The χ² statistic, derived from the residuals (differences between the original and estimated data), provides a complementary measure of model goodness of fit. A χ² value close to 1.0 indicates that the observed and predicted data distributions are in good agreement, whereas larger deviations suggest potential model bias or poor estimation capability.

Finally, the MSE was calculated to assess the average deviation between estimated and actual dose values across all structures. MSE was chosen because it emphasizes larger dose prediction errors by squaring deviations, making it more sensitive to clinically meaningful outliers than the mean absolute error. This is particularly relevant in radiotherapy planning, where larger dose discrepancies may have greater clinical impact.

Together, these parameters were used to evaluate model accuracy, detect potential overfitting, and ensure the robustness and reliability of the KBP models before clinical application.

Model validation was performed using 10 independent test plans for each laterality-specific model (left- and right-sided) and 20 independent plans for the combined model. An open-loop approach, in which the training and validation datasets are completely independent, was employed to avoid prediction bias and reduce the risk of overfitting.

Dose comparisons and data analysis

The KBP_LT_ and KBP_RT_ plans were compared with their respective clinical plans, while the KBP_c_ plans were evaluated against both the clinical plans and the laterality-specific KBP models. All KBP plans were normalized to the same target volume coverage as the corresponding clinical plans to ensure a fair and consistent comparison.

For target evaluation, the following DVH parameters were assessed: PTVTotal V95%, Dmax, PTVAxilla V95%, PTVChestwall V95%, PTVIMN V90%, and PTVSupraclavicular V95%. Total target coverage was also assessed based on ICRU 83 [15] reporting standards. The doses received by 2%, 5%, 95%, and 98% of the PTV volume (D_2%_, D_5%_, D_95%_, and D_98%_, respectively) were determined, where D_2%_ and D_5%_ represent near-maximum doses (potential hot spots), and D_95%_ and D_98%_ represent near-minimum doses, reflecting target coverage. These values were reported as relative doses.

Two conformity indices (CIs) were calculated to evaluate the degree of dose conformity to the PTVTotal. The first, CI100%, represents conformity of the prescription isodose and was defined according to the RTOG guidelines [16]:

$CI_{100\%} = \frac{V_{TV100\%}}{V_{PTV}},$

where V_TV100%_ is the treated volume enclosed by a 100% isodose surface, and V_PTV_ is the PTV. A CI_100%_ = 1.0 indicates ideal conformity, values > 1.0 reflect unnecessary irradiation of surrounding tissue, and values < 1.0 indicate under coverage of the target. Ideal CI values can be difficult to achieve for plans with irregularly shaped target volumes [17,18]. To characterize low-dose conformity, the CI_50%_ was also calculated using the same definition but referencing the 50% isodose line:

$CI_{50\%} = \frac{V_{TV50\%}}{V_{PTV}}$

Low CI_50%_ values indicate a steeper dose fall-off and reduced low-dose spillage to normal tissues. Dose uniformity within the PTVTotal was quantified using the ICRU 83 definition of the homogeneity index (HI): 

$HI = \frac{D_{2\%} - D_{98\%}}{D_{50\%}},$

where D_2%_, D_50%_, and D_98%_ are the doses received by 2%, 50%, and 98% of the PTV, respectively. Lower HI values indicate improved dose homogeneity within the target, with an ideal value of zero corresponding to a perfectly uniform dose distribution.

The DVH parameters used to evaluate the OARs are Heart Dmean, Lung Ipsilateral V65%, Lung Ipsilateral V33, Lung Contralateral V15%, Lung Contralateral V15%, Breast Contralateral V10%, Esophagus Dmax, Spinal Cord Dmax, and Thyroid Dmean in cGy.

The delivery efficiency of KBP-generated plans was evaluated by calculating the IMRT factor for each plan and comparing it with the corresponding clinical plans. The IMRT factor is defined as the ratio of the total MUs required to deliver a plan to the prescribed dose per fraction (in cGy). A duty factor below five is generally considered desirable, as it indicates shorter treatment delivery times, reduced MLC modulation complexity, and higher IMRT QA passing rates [19,20]. A two-tailed t-test was performed to assess the statistical significance of aggregate differences across all comparison metrics, with p < 0.05 considered indicative of statistical significance.

Physician blind review

Finally, a blind review was performed by a radiation oncologist to assess the clinical quality of the treatment plans. A total of 60 plans were evaluated, including 10 left-sided KBP plans, 10 right-sided KBP plans, 20 combined KBP plans generated using the KBP_c_ model, and their 20 corresponding manually generated clinical plans. The physician was blinded to plan origin and asked to assess to determine clinical acceptability and to indicate a preference between each paired KBP and clinical plan, with a “tie” option available when no clear preference could be made.

## Results

**Figure 1:**
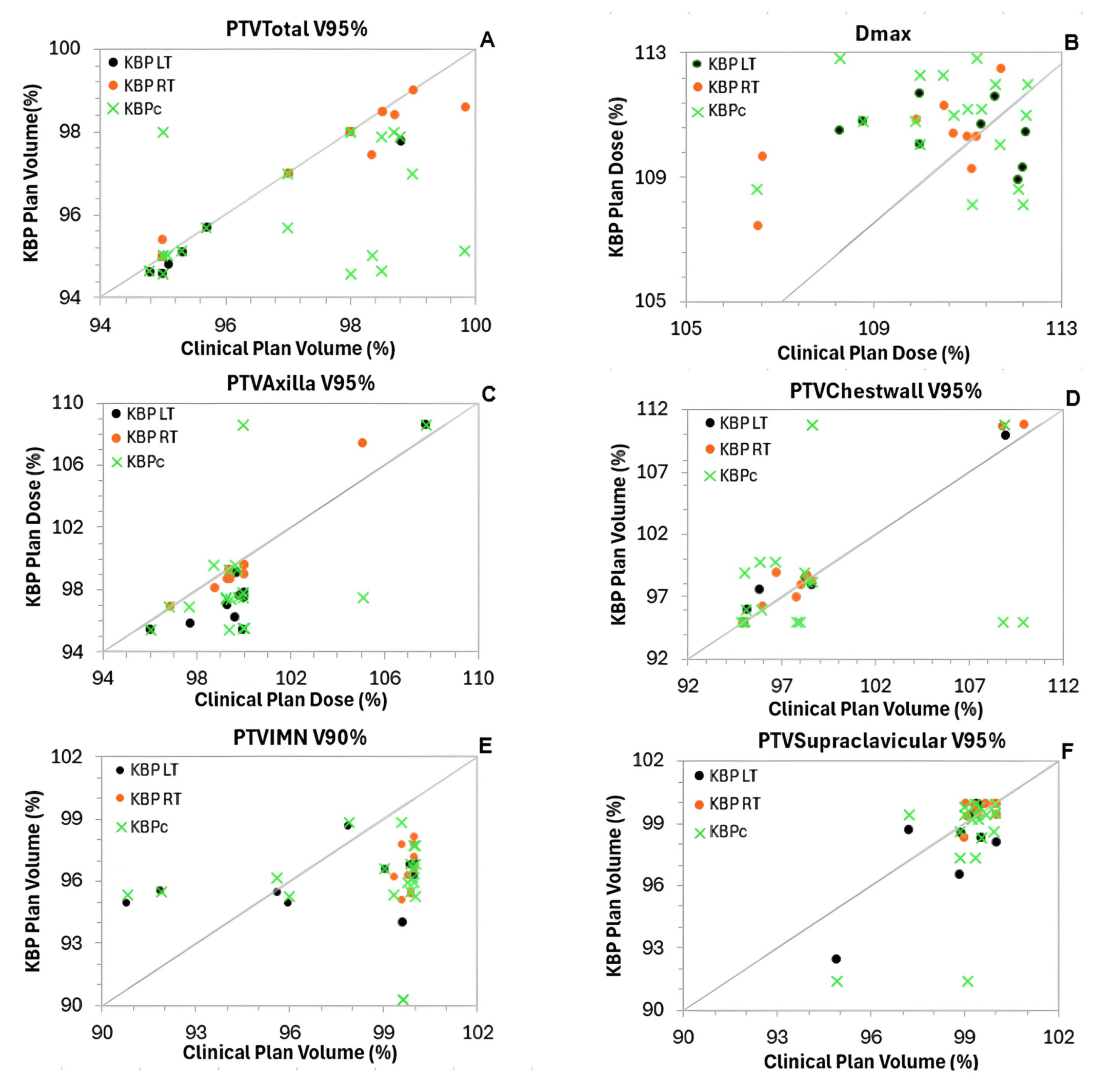
Scatter plots of DVH parameters for PTVs comparing clinical plans (x-axis) with the three KBP models (y-axis) (A) Total planning volume (PTVTotal) V95%, (B) Maximum dose (Dmax), (C) Axillary nodes PTV (PTVAxilla) V95%, (D) Chestwall PTV (PTVChestwall) V95%, (E) Internal mammary nodes PTV (PTVIMN) V90%, and (F) Supraclavicular nodes PTV (PTVSupraclavicular) V95%. Vx%: percentage of the PTV receiving at least x% of the prescribed dose. Points along the diagonal line represent cases where the KBP plans match the clinical (manual) plans. Points below the equality line indicate volume or dose reductions achieved by the KBP models for the corresponding metrics and patients. DVH, dose-volume histogram; KBP, knowledge-based planning; PTV, planning target volume

**Figure 2:**
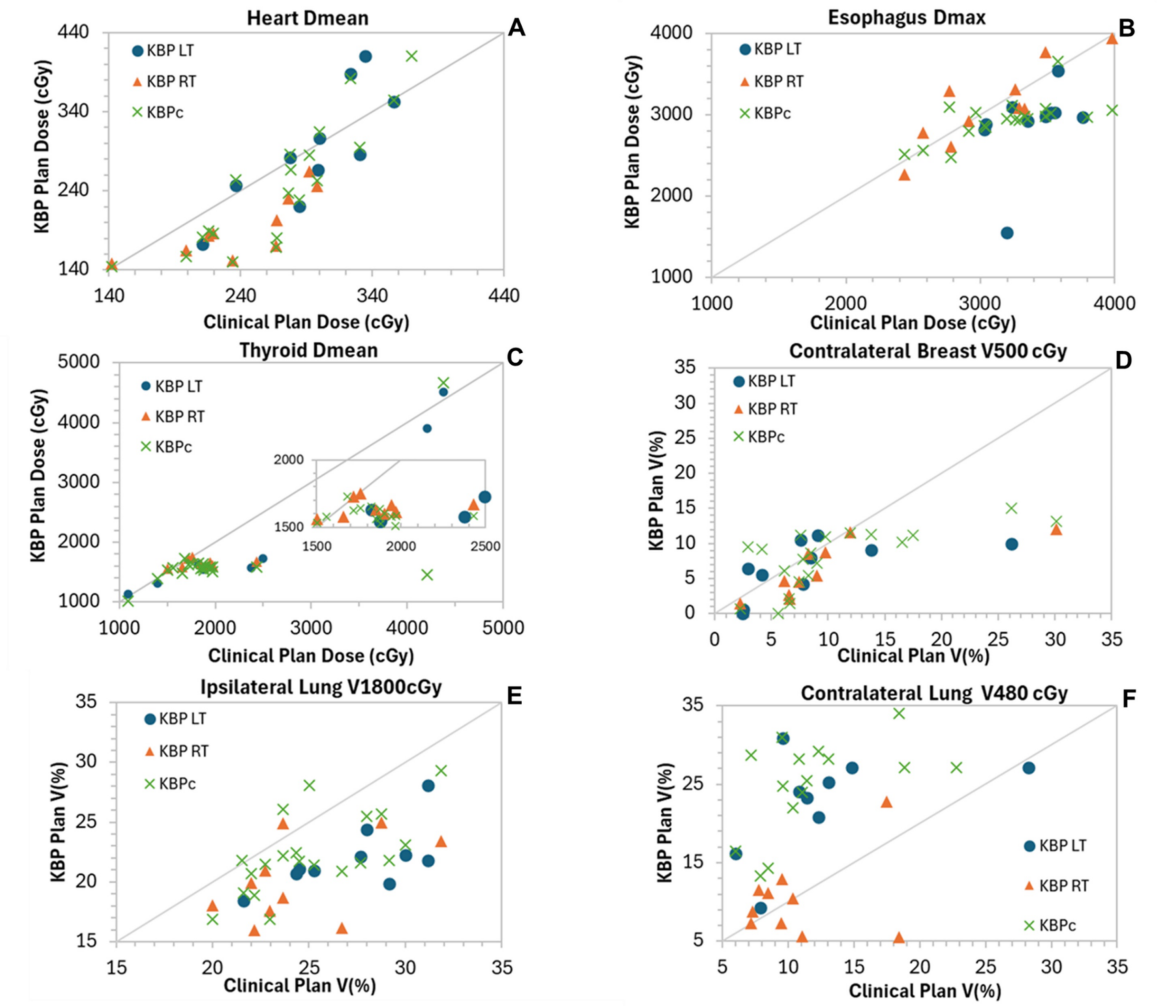
Scatter plots of DVH parameters for key OARs comparing clinical plans (x-axis) with the three KBP models (y-axis) (A) Heart mean dose (Dmean), (B) Esophagus maximum dose (Dmax), (C) Thyroid mean dose (Dmean), (D) Contralateral Breast V500cGy, (E) Ipsilateral Lung V1800cGy, and (F) Contralateral Lung V480cGy. Vx cGy: percentage of the organ volume receiving at least x cGy. Points along the diagonal line represent cases where the KBP plans match the clinical (manual) plans. Points below the equality line indicate dose reductions achieved by the KBP models for the corresponding OARs and patients. DVH, dose-volume histogram; KBP, knowledge-based planning; OAR, organ at risk

Comparisons of DVH indices between the KBP_LT_ and KBP_RT_ plans with their respective clinical plans are summarized in Table 3, while Table 4 presents the comparisons between the combined KBP model and the clinical plans. Figure 1 shows scatter plots for PTVs, while Figure 2 shows scatter plots for key OARs, illustrating the dosimetric performance of the three KBP models relative to the clinical (manual) plans and each other.

**Table 3:** Model training results for LT, RT, and combined KBP models Low OAR MSE values (<0.05) indicate high predictive accuracy. R² values of 0.2-0.9 reflect variability in predictive strength without overfitting. X² values of ~1 confirm that the observed and predicted data distributions are in good agreement. KBP, knowledge-based planning; LT, left side; MSE, mean squared error; OAR, organ at risk; RT, right side

OAR	KBP_LT_			KBP_RT_			KBP_c_		
	R²	χ²	MSE	R²	χ²	MSE	R²	χ²	MSE
Heart	0.44	1.05	0.02	0.24	1.02	0.02	0.54	1.03	0.02
Lung ipsilateral	0.29	1.06	0.02	0.69	1.08	0.02	0.81	1.02	0.02
Lung contralateral	0.24	1.07	0.02	0.41	1.07	0.02	0.75	1.03	0.02
Breast contralateral	0.74	1.2	0.02	0.36	1.08	0.02	0.81	1.02	0.02
Esophagus	0.88	1.1	0.02	0.57	1	0.02	0.73	1.07	0.02
Spinal cord	0.48	1.08	0.02	0.34	1.08	0.02	0.22	1.04	0.02
Thyroid	0.45	1.1	0.02	0.75	1	0.02	0.48	1.07	0.02

**Table 4:** Summary of dosimetric metrics for clinical plans and KBP left- and right-sided plans, along with their comparisons Values represent the mean ± SD across the 10 patients used to validate the models. Vx%: percentage of the structure’s volume receiving at least x% of the prescribed dose KBP, knowledge-based planning

Structures	Clinical LT	KBP_LT_	KBP_LT_ - clinical LT	p_KBPLT_	Clinical RT	KBP_LT_	KBP_LT_ - clinical RT	pKBP_RT_
PTVTotal (V95% ≥ %)	96.3 ± 0.5	96 ± 0.5	-0.2 ± 0.1	0.07	97.8 ± 0.5	97.59 ± 0.4	-0.2 ± 0.2	0.66
Dmax (≤ %)	111 ± 0.4	111 ± 0.4	0.11 ± 0.7	0.8	110 ± 0.6	110.5 ± 0.6	0.5 ± 0.5	0.41
PTVAxilla (V95% ≥ %)	99.9 ± 1.2	98.1 ± 1.2	-1.9 ± 0.5	0.03	99.8 ± 0.6	99.6 ± 0.9	-0.2 ± 0.3	0.85
PTVChestwall (V95% ≥ %)	97.2 ± 1.4	97.5 ± 1.4	0.3 ± 0.2	0.1	99.4 ± 1.7	99.9 ± 1.9	0.5 ± 0.3	0.8
PTVIMN (V90% ≥ %)	97.1 ± 0.4	96 ± 0.4	-1.1 ± 1.0	0.05	99.8 ± 0.1	96.6 ± 0.3	-3.2 ± 0.3	<0.001
PTVSupraclavicular (V95% ≥ %)	98.7 ± 0.7	98.2 ± 0.7	-0.5 ± 0.4	0.25	100.4 ± 0.9	100.4 ± 0.8	0.02 ± 0.2	0.98
Heart (mean ≤ cGy)	295.2 ± 23.4	293.4 ± 23.4	-1.8 ± 14.4	0.9	241.1 ± 15.4	195 ± 12.6	-46.1 ± 9.3	0.005
Lung ipsilateral (V65% ≤ %)	61.8 ± 2.3	58.1 ± 2.3	-3.2 ± 2.3	0.22	62.1 ± 0.6	57.6 ± 4.3	-4.5 ± 4.5	0.32
Lung ipsilateral (V35% ≤ %)	27.3 ± 0.8	21.9 ± 0.8	-5.3 ± 0.8	<0.001	24.4 ± 1.1	20.1 ± 1.1	-4.4 ± 1.1	0.002
Lung contralateral (V15% ≤ %)	11.9 ± 2.1	22.1 ± 2.0	10.1 ± 1.9	<0.001	10.7 ± 1.3	10.3 ± 1.6	-0.4 ± 1.7	0.83
Breast contralateral (V10% ≤ %)	8.5 ± 1.2	6.5 ± 1.25	-1.9 ± 1.8	0.31	9.8 ± 2.4	6.19 ± 1.2	-3.6 ± 1.7	0.02
Esophagus (max)	3377 ± 161	2878 ± 161	-499 ± 147	0.01	3083 ± 149	3105 ± 160	22 ± 79	0.9
Spinal cord (max)	1259 ± 172	1279 ± 172	20 ± 82	0.81	1403 ± 30	1383 ± 35	-20 ± 37	0.58
Thyroid (mean)	2340 ± 367	2044 ± 367	-296 ± 96	0.01	1867 ± 88	1647 ± 22	-220 ± 86	<0.001

Compared with manual plans, the KBP_LT_ plans achieved significantly lower doses to the esophagus (−499 ± 147 cGy) and thyroid (−296 ± 96 cGy), both statistically significant (p < 0.05). KBP_LT_ also reduced doses to all other OARs, with the exceptions of the spinal cord (+20 ± 82 cGy) and contralateral lung V35% (+10.1 ± 1.9 cGy). The difference in spinal cord was not statistically significant (p > 0.05), while the increase in contralateral lung V35% was statistically significant; however, all OAR dose constraints were still met.

For target volumes, comparing the KBP_LT_ plans with manual plans, only the IMN coverage (-1.1 ± 1.0%) and axilla target coverage (-1.9 ± 0.5%) differences were statistically significant. Despite this reduction, the coverage remained above the planning objectives, as most clinical plans had overachieved the target coverage.

A similar pattern was observed for the targets of the KBP_RT_ model, with the only statistically significant difference in target coverage found for the PTV_IMN. For OARs, the greatest dose reductions were achieved for the thyroid mean dose (-220 ± 86 cGy) and the mean heart dose (-46.1 ± 9.3 cGy), both statistically significant. Reductions were also seen across all other OARs, with statistically significant improvements noted for the ipsilateral lung V35% and the contralateral breast V10%.

For the combined KBP model, a similar overall pattern was observed, with clinically meaningful and statistically significant improvements in several key metrics. Among the targets, a statistically significant reduction was observed for PTV_IMN (-3.0 ± 0.6%, p = 0.01), while coverage for the remaining target volumes showed minor, non-significant differences compared to the clinical plans. 

For OARs, the most notable and statistically significant dose reductions achieved with the KBP_c_ model were for the heart mean dose (-55.9 ± 9.2 cGy, p = 0.02), the esophagus maximum dose (-400.0 ± 70.2 cGy, p < 0.001), the spinal cord maximum dose (-152.6 ± 44.5 cGy, p = 0.48), and the thyroid mean dose (-79.9 ± 138.7 cGy, p = 0.03). Significant dose sparing was also observed for the ipsilateral lung (V35%) (-2.5 ± 0.6%, p < 0.001), while the contralateral lung (V15%) and contralateral breast (V10%) exhibited slight increases (5.4 ± 1.8% and 0.1 ± 1.2%, respectively), both reaching statistical significance. 

**Figure 3:**
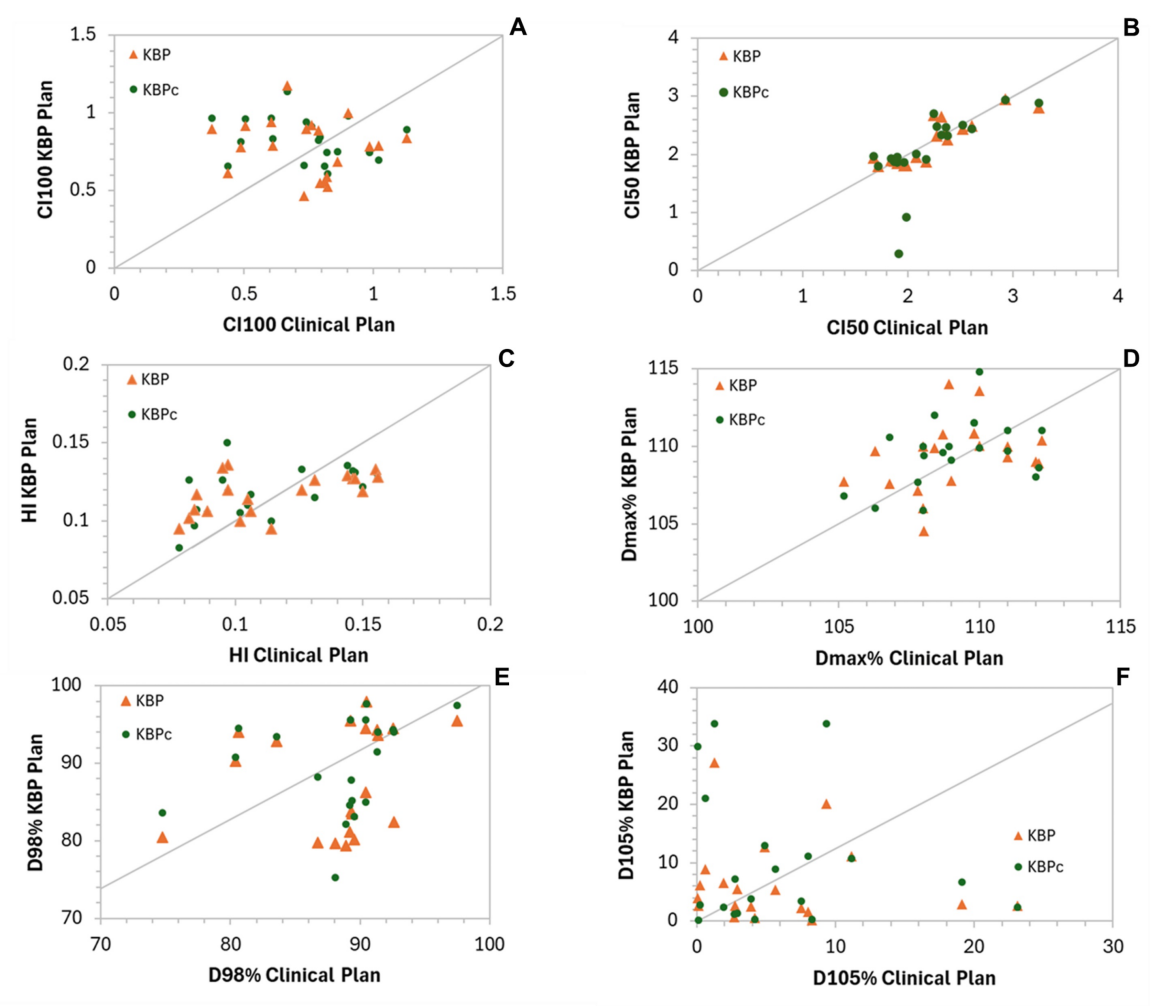
Scatter plots of plan quality and dosimetric indices comparing laterality-specific KBP plans with clinical plans Clinical plans are shown on the x-axis, and KBP model plans are shown on the y-axis. (A) CI of the 100% isodose line (CI100), (B) CI of the 50% isodose line (CI50), (C) HI, (D) Maximum Dose (Dmax%), (E) Dose received by the 98% of the PTV (D98%), and (F) Dose received by the 105% of the PTV (D105%). Points along the diagonal line indicate identical performance between the KBP and clinical plans. Points below the equality line represent cases where the KBP models achieved improved plan quality, such as better conformity or homogeneity, compared with the manual plans. CI, conformity index; HI, homogeneity index; KBP, knowledge-based planning

Scatter plots of the plan quality indices comparing the laterality-specific KBP plans and the combined KBP model with the corresponding clinical plans are shown in Figure 3. Overall, 60% of the KBP plans, both side-specific and combined, were more conformal to the PTVTotal than the clinical plans. Lower CI₅₀% values indicate a steeper dose fall-off and reduced low-dose spillage to normal tissues. As shown in Figure 3, nearly all KBP plans achieved lower CI₅₀% values than their clinical counterparts, with only two cases in the laterality-specific models and three cases in the combined KBP model showing the opposite trend. In Table 4. The model training results are summarized for the key OAR.

Half of the KBP plans demonstrated greater homogeneity (lower HI values) than their corresponding clinical plans. The mean D98% values for the clinical, laterality-specific KBP, and combined KBP plans were 88.3 ± 1.1 cGy, 88.5 ± 1.4 cGy, and 89.7 ± 1.4 cGy, respectively, with 60% of the laterality-specific and 65% of the combined KBP plans achieving higher D98% values than the clinical plans. On average, KBP plans increased the D105% by 0.4 ± 0.1 cGy, while the combined KBP increased it by 3.8 ± 0.8 cGy.

The average Dmax values were comparable across all plan types: 109.8 ± 0.4% for clinical, 110.7 ± 0.5 cGy for KBP, and 111.5 ± 0.5 cGy for KBP_c_, with nine out of 20 laterality-specific and combined KBP plans showing slightly higher Dmax values than the clinical plans, though all remained below the 115% maximum dose constraint. It is worth noting that KBP plans tended to increase Dmax; therefore, body dose constraints were applied in a secondary optimization run to mitigate this effect.

**Figure 4:**
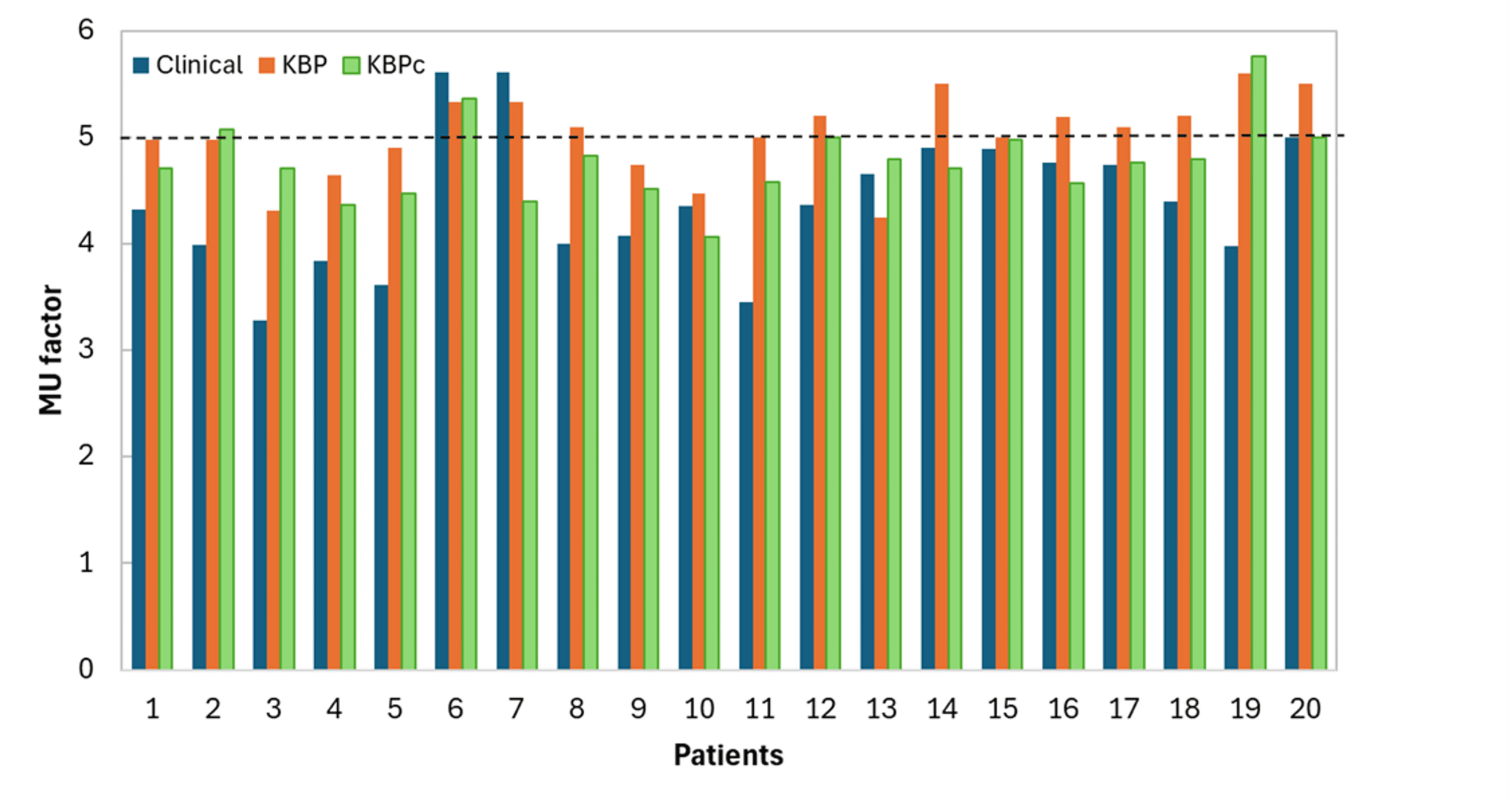
MU factor comparison KBP, knowledge-based planning; MU, monitor unit

An increase in the MU factor was observed for all KBP plans (Figure 4); therefore, an additional MU constraint was introduced during optimization to limit excessive modulation. Although higher MU values are generally associated with increased plan complexity, they are also expected for large, irregular targets such as the chest wall and nodal volumes. As shown in Figure 4, most KBP_c_ plans achieved MU factors below 5 (85%), indicating acceptable plan efficiency and modulation, whereas 50% of the laterality-specific KBP plans exceeded an MU factor of 5. Notably, two clinical plans already had MU factors above 5, and the corresponding KBP plans were able to reduce the MU values for both cases.

For cases 3, 5, 11, and 19, the MU factors for the clinical plans were 3.2, 3.5, 3.5, and 3.9, respectively, whereas the corresponding KBP plans had MU factors of 4.3, 4.9, 5.0, and 5.5. This increase in MU, even with the secondary constraint to control modulation, indicates that KBP plans generally produce higher complexity in terms of modulation. However, it also suggests that these specific clinical cases may have had additional room for modulation during manual planning, allowing the KBP models to explore and improve dose distributions.

Blind review

For the left-sided cases, the physician preferred the KBP_LT_ plan in three out of 10 cases and the corresponding clinical plan in three out of 10 cases. The rationale behind the physician’s selections was driven primarily by achieving a mean heart dose below 300 cGy and maintaining adequate IMN coverage. A tie between the KBP_LT_ and clinical plans was recorded in one case, while in one case, the physician indicated a tie between the KBP_LT_ and KBP_c_ plans. In the remaining two cases, all plans (KBP_LT_, KBP combined, and clinical plans) were rated as equivalent.

For the right-sided cases, the physician generally favored the KBP-based plans, preferring either the KBP_RT_ or KBP_c _plans in eight out of 10 cases. The physician’s selections were driven mainly by achieving a mean heart dose below 300 cGy, reducing the ipsilateral V20, and reducing the mean lung dose. Specifically, five cases were rated in favor of the KBP_RT_ or KBP_c_ plans, two cases were preferred as KBP_RT_, and one case was preferred as KBP_c_. In the remaining two cases, all plans were rated as equivalent, including the clinical plans.

It is worth noting that even in the cases where the physician preferred the clinical plan, all KBP-derived plans were deemed clinically acceptable, except for one KBP_LT_ plan, which was considered unacceptable due to its higher mean heart dose and ipsilateral lung dose.

## Discussion

In this study, three KBP models were developed for left-, right-, and combined-sided chest wall irradiation with regional lymph node coverage using the VMAT technique. All models were generated and validated using data from our institution. The laterality-specific models were trained with 47 left-sided and 44 right-sided cases and validated with 10 independent clinical plans each, while the combined model incorporated all 91 cases for training and was validated with 20 clinical plans (10 left-sided and 10 right-sided). Although existing literature generally recommends training KBP models with a relatively large number of cases and the use of distinct training and validation sets [21], these recommendations are based on prior KBP studies that systematically evaluate model performance across a wide range of training set sizes rather than formal guidelines. For example, Boutilier et al. [21] investigated datasets ranging from 10 to 200 cases and found that the minimum number of cases required for stable model performance depends on both the specific KBP approach and the predicted endpoint. In the present study, the dataset size lies toward the lower end of the ranges examined in prior work; however, acceptable clinical performance with smaller datasets has been reported [22]. Notably, Kaderka et al. [22] demonstrated that KBP models trained on a limited number of cases (31 validation plans) can still achieve clinically acceptable outcomes, simulating situations where data availability is constrained. Together, these findings support the suitability of the dataset used in this work for model development and validation.

The statistical metrics, including the mean square error and coefficient of determination, indicated stable model behavior without signs of overfitting. These values were consistent with those reported in prior publications [12], supporting the reliability of our models. Independent validation was carried out with 10 new cases for each laterality-specific model and 20 for KBP_c_, confirming that the KBP-generated plans produced dosimetric results comparable to manually optimized clinical plans. Most KBP-generated plans demonstrated small but consistent improvements in selected OARs, particularly the thyroid, esophagus, and mean heart doses, as reported in the Results section. These improvements may be related to the iterative model refinement performed during training, which accounted for variability in clinical optimization practices. Specifically, the training dataset included plans in which certain organs (e.g., the thyroid) were not explicitly optimized or were defined using different optimization structures. Incorporation of this variability during iterative model refinement contributed to the observed improvements in OAR sparing.

KBP planning substantially reduced the time required for plan generation, with each case taking about 15 minutes from optimization to final dose calculation, compared with the two to three hours typically needed for manual planning. This significant time efficiency allows planners to focus on patient-specific optimization rather than baseline plan creation. Furthermore, KBP models enable planners with varying levels of experience to produce high-quality, consistent plans, a finding supported by previous studies [23].

A few instances of unmet dose constraints were observed. In the left-sided model, one plan slightly exceeded the mean heart dose, while minor deviations were noted for the ipsilateral (V1800cGy < 35%) and contralateral lung (V480cGy < 15%) constraints. The right-sided model had one case exceeding the contralateral lung constraint, with similar occurrences observed in the combined KBP model. Overall, the laterality-specific KBP models demonstrated slightly better performance than the combined model in meeting clinical dose limits.

These findings emphasize the continued need for careful plan evaluation before clinical approval. In cases where the initial RP-generated plan did not meet a constraint, a secondary optimization run with increased priority on the violated objective successfully improved plan quality and restored compliance with clinical goals. For example, when the ipsilateral and contralateral lung constraints were exceeded, a control structure encompassing both lungs, excluding the PTV with a 2-3 cm margin, was introduced. This strategy improved distribution and ensured that all constraints were satisfied. These results suggest that minor manual refinement may still be necessary for cases of higher complexity, such as those with larger tumor volumes, proximity to critical organs, or unusual anatomical variations. Nevertheless, the KBP-generated plans presented here can serve as efficient starting points for clinical plan generation and as standardized baselines for comparative studies, such as photon versus proton therapy or VMAT versus 3D conformal techniques, providing a consistent and reproducible framework for evaluating plan quality.

The physician’s blind review demonstrated that the KBP models consistently produced treatment plans of comparable or superior quality to manually generated clinical plans. Overall, the physician tended to prefer KBP-generated plans or rated them as equivalent to clinical plans, indicating that the KBP models achieved a clinically acceptable level of plan quality and could be confidently implemented in routine practice.

Qualitative feedback from the reviewing physician supported this observation, noting that all evaluated plans were clinically acceptable and that the differences among them were minimal. The physician emphasized that such variations were likely within the normal range of planning uncertainty and unlikely to translate into meaningful clinical differences over the course of treatment. The review focused primarily on key dosimetric parameters, including ipsilateral lung V2000 cGy, mean ipsilateral lung dose, mean heart dose, and IMN coverage, which are the primary clinical considerations in breast and RNI. Doses to contralateral structures were considered of secondary importance and showed minimal variation between plans. The overall preference toward KBP_LT/RT_ or KBP_c_ models, along with frequent ties between KBP and manual plans, suggests that the automated approach can generate plans that are both efficient and clinically robust.

Despite these encouraging results, some limitations of this study should be acknowledged. The models were created and tested within a single institution, which may limit their generalizability due to institutional planning techniques and priorities. However, the dataset encompassed an extended time span and included plans generated by multiple planners, introducing diversity in clinical practice and optimization strategies. In addition, the blinded review was conducted by a single physician, which may introduce subjective bias as clinical judgment can vary among reviewers. Conversely, the involvement of a single highly experienced radiation oncologist minimizes inter-observer variability and reduces the impact of subjective differences on the analysis. As discussed in the Results section, an increase in the MU factor was observed in most laterality-specific KBP plans, which may represent a potential limitation of the presented models. Higher MU values can indicate increased plan complexity, which could have implications for plan deliverability and quality assurance. A useful direction for future work would be to systematically evaluate the MU factor across a larger cohort of laterality-specific KBP plans to identify potential patterns of increase and to assess the impact on deliverability, for example, through IMRT QA measurements. Despite these limitations, our findings indicate that well-designed KBP models, even when trained on moderately sized datasets, can produce clinically acceptable, efficient, and reproducible treatment plans. These results support the broader integration of KBP in clinical workflows to enhance planning consistency and efficiency.

## Conclusions

This study developed and evaluated three KBP models for VMAT-based chest wall and regional lymph node irradiation. The laterality-specific and combined KBP models generated plans comparable in quality to manually created clinical plans, while maintaining consistent target coverage and favorable sparing of OARs. These findings highlight the potential of KBP to enhance planning efficiency and reproducibility in complex chest wall cases involving nodal regions. While minor manual refinements, such as an additional optimization run, may occasionally be required, particularly for the combined KBP model, the results support the integration of KBP into clinical workflows as robust starting points for planning, improving standardization without compromising plan quality.
